# Determining the Impact of Thickened Liquids on Swallowing in Patients Undergoing Irradiation for Oropharynx Cancer

**DOI:** 10.1177/01945998211010435

**Published:** 2021-05-04

**Authors:** Carly E. A. Barbon, Douglas B. Chepeha, Andrew J. Hope, Melanie Peladeau-Pigeon, Ashley A. Waito, Catriona M. Steele

**Affiliations:** 1 Rehabilitation Sciences Institute, University of Toronto, Toronto, Canada; 2 Toronto Rehabilitation Institute–University Health Network, Swallowing Rehabilitation Research Laboratory, Toronto, Canada; 3 Department of Otolaryngology–Head and Neck Surgery, University of Toronto, Toronto, Canada; 4 Department of Radiation Oncology, University of Toronto, Toronto, Canada

**Keywords:** dysphagia, head and neck cancer, texture modification

## Abstract

The current standard for the treatment of oropharynx cancers is radiation therapy. However, patients are frequently left with dysphagia characterized by penetration-aspiration (impaired safety) and residue (impaired efficiency). Although thickened liquids are commonly used to manage dysphagia, we lack evidence to guide the modification of liquids for clinical benefit in the head and neck cancer population. The objective of this study was to assess the impact of slightly and mildly thick liquids on penetration-aspiration and residue in 12 patients with oropharyngeal cancer who displayed penetration-aspiration on thin liquid within 3 to 6 months after completion of radiotherapy. Significantly fewer instances of penetration-aspiration were seen with slightly and mildly thick liquids as compared with thin (*P* < .05). No differences were found across stimuli in the frequency of residue. Patients with oropharyngeal cancers who present with post–radiation therapy dysphagia involving penetration-aspiration on thin liquids may benefit from slightly and mildly thick liquids without risk of worse residue.

A patient’s ability to eat by mouth is affected by radiation therapy (RT) for oropharynx cancers.^1,2^ RT spares critical structures but contributes to functional impairments of swallowing safety (penetration-aspiration; ie, food/liquid entering the airway) and efficiency (pharyngeal residue). Maintaining oral intake is encouraged during RT to avoid negative consequences of muscle disuse.^3,4^ However, aspiration occurs in 18% to 33% of patients with head and neck cancer (HNC) over the first 2 years following chemoradiation. Aspiration is linked to aspiration pneumonia and associated mortality.^5-17^ Two systematic reviews have concluded that thicker liquids reduce aspiration risk in the HNC population^18,19^; however, the evidence is limited to extremely thick liquids,^20^ which may lead to increased residue.^16,21-25^ We explored the effectiveness of smaller degrees of thickening for improving swallowing safety without exacerbating residue, using slightly and mildly thick liquids as defined by the International Dysphagia Diet Standardisation Initiative.^26^

## Methods

Human subjects research approval was provided by the University Health Network Research Ethics Board (UHN-CAPCR 16-5190). We enrolled 12 men (mean, 63.3 years; range, 49-78) in the 3- to 6-month time frame post-RT for primary T1-3, N0-N2c cancers of the oropharynx^27^ (**Table 1**). Participants underwent a videofluoroscopic swallowing study (VFSS) with 20% w/v liquid barium (E-Z-Paque powdered barium; Bracco) prepared in thin, slightly thick, and mildly thick liquid consistencies with commercially available thickeners (Resource ThickenUp and ThickenUp Clear; Nestlé Health Science).^28^ The protocol included 15 naturally sized sips, beginning with thin liquid. If penetration-aspiration was not detected with thin liquid, the protocol was terminated. When material entered the larynx (Penetration-Aspiration Scale [PAS]^29^ score ≥3), the protocol proceeded to the next level of thickness. For safety, the protocol was terminated after 4 observations of penetration-aspiration.

**Table 1.** Study Participant Demographics.^a^

Age, y	Site	Stage	Dose, cGy	Chemotherapy	HPV status	Days between RT and VFSS
78	Base of tongue	T2, N2b	5200	NA	+	105
67	Base of tongue	T3, N2b	7000	Cisplatin	−	111
66	Base of tongue	T2, N2b	7000	Cisplatin	+	113
61	Base of tongue	T3, N2b	7000	Cisplatin^b^	+	113
58	Oropharynx	T0, N2b	7000	Cisplatin^b^	+	151
49	Soft palate	T3, N2b	7000	Cisplatin	−	140
77	Soft palate	T1, N0	6000	NA	−	134
61	Right tonsil	T2, N1	7000	NA	+	92
75	Base of tongue	T1, N2b	7000	NA	+	89
58	Right tonsil/base of tongue	T2, N2c	7000	Cetuximab^b^	+	111
60	Left tonsil	T1, N2b	7000	Cisplatin (high dose)	+	135
51	Base of tongue	T3, N2b	7000	Cisplatin	+	203

Abbreviations: HPV, human papillomavirus; NA, not applicable; RT, radiation therapy; VFSS, videofluoroscopic swallowing study.

a All patients were male.

b Full chemotherapy protocol not completed.

**Figure 1.**
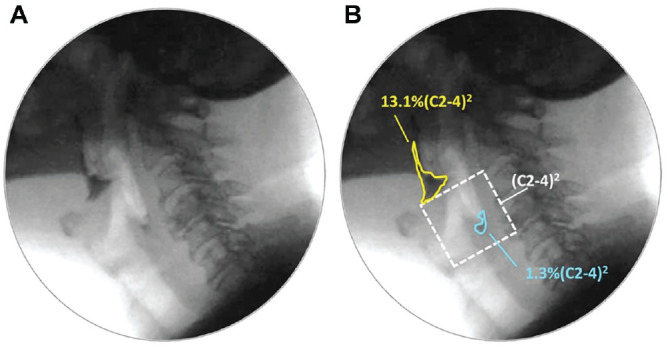
Pixel-based tracings of residue. (a) Pharyngeal residue. (b) Residue is traced and compared with an anatomic reference scalar (squared length of the C2-4 cervical spine), shown with white dashed lines.

VFSS recordings were randomly assigned for independent duplicate rating according to the ASPEKT Method (Analysis of Swallowing Physiology: Events, Kinematics and Timing).^14^ PAS scores were converted to binary ratings of safe versus “unsafe” (PAS <3 vs ≥3).^29^ Anatomically scaled pixel-based measures of pharyngeal residue (%[C2-4]^2^)^30^ were converted to binary ratings of efficient versus “at risk” (residue >1%[C2-4]^2^; see **Figure 1**).^31^ Nonparametric Friedman tests were used to compare the frequencies of safe/unsafe and efficient/at-risk swallows by consistency.

## Results

During the VFSS, 6 of 12 participants (50%) had penetration-aspiration on thin liquids and continued to the thickened liquid portion of the protocol. Subsequent blinded rating identified 3 more participants with PAS scores ≥3 for thin liquid; however, these participants did not complete the thickened liquid trials. **Table 2** displays frequency data for safe/unsafe and efficient/at-risk boluses by consistency.

**Table 2.** Boluses Classified as Safe/Unsafe and Efficient/At Risk by Consistency. ^a^

	Penetration-Aspiration Scale		Residue	
Stimulus	<3 (safe)	≥3 (unsafe)	≤1%(C2-4)^2^ (efficient)	>1%(C2-4)^2^ (at risk)
Thin	60.70	39.30	53.80	46.20
Slightly thick	81.80	18.20	16.70	83.30
Mildly thick	81.80	18.20	16.70	83.30
Total	75.50	24.50	27.80	72.20

a Values are presented as percentages.

The frequency of unsafe PAS scores differed significantly by consistency, χ^2^(2) = 8.667, *P* < .005. Significantly fewer penetration-aspiration events (*P* < .05) were seen with slightly thick and mildly thick liquids as compared with thin. The frequency of unsafe swallows did not differ between slightly and mildly thick liquids. With respect to efficiency, there were no significant differences across consistency.

## Discussion

This study explored the impact of thickened liquids on swallowing in a homogeneous sample of patients who had oropharynx cancer with penetration-aspiration on thin liquids. The data corroborate previous evidence that impaired swallow safety is common post-RT,^4,8,16,32-34^ with the majority of participants demonstrating penetration-aspiration on thin liquids (9/12, 75%). This points to risk with oral consumption of liquid nutritional supplements in the subacute phase postradiation given the associated risk for pneumonia, particularly because sensory deficits may obscure clinical signs of aspiration.^35,36^

Our findings concur with previous studies showing reduced penetration-aspiration with thickened liquids.^13-15,17^ In comparison with previous evidence of aspiration reduction with extremely thick liquids, which are disliked by many patients,^15^ this study shows potential to achieve safe swallowing with minimal thickening. However, pharyngeal residue was also a common finding, regardless of bolus consistency. Residue >1%(C2-4)^2^, which has been shown to double the odds of penetration-aspiration on the subsequent swallow,^37^ was seen in almost half (46%) of the thin liquid bolus trials in this study.

It is important to determine the mechanisms underlying unsafe and inefficient swallowing in patients with oropharynx cancer. Delayed or incomplete laryngeal vestibule closure has been identified as a primary mechanism contributing to penetration and aspiration.^38-40^ Thickened liquids allow for additional time in which to achieve laryngeal vestibule closure. Several recent studies also point to poor pharyngeal constriction being a primary mechanism behind pharyngeal residue.^39,41-44^ The pathophysiological drivers of pharyngeal function and the association to residue in radiated patients remains unknown.

This study is not without limitations. Our efforts to recruit a homogeneous group of patients in terms of cancer location and severity resulted in a small study sample. The requirement that impaired safety be identified during VFSS on thin liquids also resulted in limited available data for thickened liquids. Several instances of penetration-aspiration on thin liquids were missed by the clinicians present during the VFSS and were detected only during postexamination blinded review.

## Conclusions

We investigated the impact of thickened liquids in patients with oropharynx cancers with penetration-aspiration on thin liquids. Penetration-aspiration reduction can be achieved with minimal degrees of thickening, starting with a slightly thick consistency. This is important, given that patients may be more accepting of regular beverages such as smoothies that are naturally thick. This study suggests that residue is common but not necessarily worsened with slightly or mildly thick liquids. Further research is recommended regarding the interaction between mechanisms of impairment and liquid thickness in the broader HNC population.
